# Biliary Reconstruction in Liver Transplantation with Primary Sclerosing Cholangitis: Roux-en-Y Hepaticojejunostomy or Duct-to-Duct Anastomosis?

**DOI:** 10.3390/jcm14238518

**Published:** 2025-12-01

**Authors:** Peter T. Dancs, Mira C. Pohlmann, Jan Bednarsch, Jassin Rashidi-Alavijeh, Sophia M. Schmitz, Andreas Kroh, Florian Ulmer, Florian W. R. Vondran, Ulf P. Neumann, Dieter P. Hoyer, Daniel Heise

**Affiliations:** 1 Department of General, Visceral, Vascular and Transplantation Surgery, University Hospital Essen, 45147 Essen, Germany; 2 Department of Gastroenterology, Hepatology and Transplantation Medicine, University Hospital Essen, 45147 Essen, Germany; 3 Department of General, Visceral, Pediatric and Transplantation Surgery, University Hospital RWTH Aachen, 52074 Aachen, Germany

**Keywords:** liver transplantation, primary sclerosing cholangitis, biliary reconstruction

## Abstract

**Background/Objectives**: Biliary reconstruction in liver transplantation (LT) for primary sclerosing cholangitis (PSC) is controversial. A Roux-en-Y hepaticojejunostomy (HJ) is associated with fewer anastomotic strictures, while a duct-to-duct reconstruction (DD) shows a decreased rate of cholangitis and preserves anatomy for endoscopy. The aim of our study was to analyze patient survival and postoperative outcomes after LT for PSC based on the type of reconstruction in two high-volume LT centers. **Methods**: We included 94 PSC patients who underwent a primary LT between 2010 and 2024. The association of biliary reconstruction with patient survival was assessed with the Kaplan–Meier method. Predictors of mortality and postoperative complications were identified via Cox and logistic regression. **Results**: In total, 42 patients received an HJ and 52 patients received a DD. There was no difference in patient survival or major complications. DD resulted in an increased number of anastomotic strictures, whereas anastomotic insufficiency, ischemia, cholangitis, and the need for revision surgery showed no difference. The choice of biliary reconstruction technique was not a predictor for mortality or major complications. **Conclusions**: Both types of biliary reconstruction are effective for PSC patients, with comparable patient survival and postoperative outcomes. Although DD is associated with an increased number of strictures, endoscopic treatment options provide a feasible solution.

## 1. Introduction

Primary sclerosing cholangitis (PSC) is a chronic progressive disease associated with the periductular fibrosis of intrahepatic and extrahepatic bile ducts, leading to strictures, decompensation, and eventually end-stage liver disease. Despite recent advances in understanding the pathogenesis of PSC, effective treatment with a proven survival benefit is still lacking. The only remaining option is liver transplantation (LT) in the case of progressing liver cirrhosis [1]. However, LT in PSC patients is hampered through an increased number of biliary complications [2], and the state-of-the-art technique for biliary reconstruction is still a matter of debate. Historic recommendations prefer a Roux-en-Y hepaticojejunostomy (HJ), based on the theory that the inflamed native duct, affected by PSC, may play a role in developing recurrent disease as well as cholangiocarcinoma. However, the absence of the sphincter of Oddi function was presumed to result in a higher rate of ascending cholangitis. In support of this empiric recommendation, an early report from the UK, including PSC patients from seven LT centers, reported superior patient and graft survival, as well as a reduced number of strictures, in patients who underwent a Roux-en-Y HJ compared with a duct-to-duct (DD) anastomosis [3].

More recent studies, however, challenge these early propositions, reporting comparable results applying a DD reconstruction. Additionally, this method has the advantage of preserved anatomy for later endoscopic interventions [4,5,6,7,8].

As seen above, there is no ultimate answer regarding which type of biliary reconstruction is superior; arguments for both techniques are partially theoretically supported with scarce data from studies with retrospective, monocentric designs including 10–60 patients each [9]. In line with this, the aim of our study was to compare patient survival and general and biliary complication rates between the two types of biliary reconstruction in patients who underwent LT for PSC in two high-volume LT centers in Germany in the Eurotransplant (ET) region.

## 2. Materials and Methods

### 2.1. Study Population

We performed a retrospective bicentric analysis, including all consecutive LTs performed with the indication of PSC from February 2010 to June 2024 at the University Hospital Essen and the University Hospital RWTH Aachen, Germany. Recipients < 18 years of age, retransplants, recipients of multiorgan grafts, and living-donor allografts were excluded from our analysis. All livers were donated by brain-dead heart-beating donors. All data were collected from our own databases as well as the ET International Foundation. The patient cohort was partly included in a previous study of ours, investigating other aspects of LT for PSC [10]. This study was conducted in accordance with both the Declarations of Helsinki and Istanbul.

### 2.2. Surgery and Immunosuppression

Organ procurements were carried out in standard fashion as defined by ET [11]. Transplantation was performed with caval replacement and end-to-end anastomoses of the hepatic artery and the portal vein. Bile duct reconstruction was carried out based on the decision of the performing surgeon, carefully assessing the native and donor ducts for dominant stenosis of the recipient duct, sufficient passage of a biliary probe to the duodenum, as well as possible incongruencies between donor and recipient ducts. If both ducts showed a nearly similar size, a duct-to-duct anastomosis was performed with a polydioxanone, absorbable, monofilament suture in interrupted or continuous technique, based on the size of the ducts. In cases of larger discrepancies in size of the native and donor ducts, dominant stenosis of the distal recipient duct, or insufficient passage through the native duct into the duodenum, a Roux-en-Y hepaticojejunostomy was performed. The anastomosis between the Roux limb and the donor bile duct was carried out with a polydioxanone, monofilament suture in interrupted technique. The afferent limb was anastomosed end-to-side with a polydioxanone, monofilament suture in continuous technique. Bypass techniques were not applied at either center. The regimen of immunosuppression was standardized utilizing intravenous corticosteroids intraoperatively, followed by a reduction scheme, calcineurin inhibitors (tacrolimus, trough level 6–8 ng/mL), and mycophenolate mofetil (0.5–1 g, twice daily). All patients were treated and observed postoperatively at an ICU.

### 2.3. Study Variables and Outcome Parameters

Patient demographics, donor variables, surgical variables, and postoperative complications were extracted via medical record reviews. Complications were assessed via the comprehensive complication index (CCI) [12], and a CCI > 75 was considered a major complication. An anastomotic stricture was defined as an isolated stricture at the biliary anastomosis, requiring endoscopic or surgical treatment. PSC recurrence was defined by clinical or histological diagnosis. An episode of cholangitis was defined by clinical diagnosis, based on symptoms, laboratory results, liver function, and imaging. Bile leakage and ischemia of the bile duct were defined as intraoperative or endoscopic diagnosis.

### 2.4. Statistics

Continuous variables are presented as means or median and standard deviation and range, as appropriate. Categorical data are depicted by frequency and percentages. Student’s *t*-test and the χ2 test were used for group comparisons. Survival analysis was performed using the Kaplan–Meier method. Cox regression analysis was performed to identify independent risk factors for overall survival. Logistic regression was utilized to determine independent risk factors for the development of postoperative complications. Variables with a *p* value < 0.200 in univariate analysis were included in a mixed multivariate regression analysis. As all patients transplanted in Aachen received an HJ, transplant centers were not involved in our multivariate analysis, albeit a significant difference between the two groups, to avoid redundance in statistics. All statistical analyses were performed using SPSS Statistics for Windows, Version 24 (IBM Corporation, Armonk, NY, USA), GraphPad Prism, Version 6.1 (GraphPad Software Inc., La Jolla, CA, USA) and Excel (Microsoft Corporation, Redmond, WA, USA). A *p* value of <0.050 was considered statistically significant, and two-tailed tests were used for all statistical analyses.

## 3. Results

### 3.1. Study Population

A total of 94 patients who underwent a primary LT for PSC between February 2010 and June 2024 were included in our study. Biliary reconstruction was performed by Roux-en-Y hepaticojejunostomy (HJ) in 42 cases, while 52 patients received a duct-to-duct anastomosis (DD). Every patient transplanted in Aachen (*n* = 21) were reconstructed with HJ. Demographic, donor, and intraoperative characteristics of both groups are shown in Table 1. Overall 90-day, 1-year, 3-year, and 5-year survival rates were 92.5%, 89.1%, 84.0%, and 82.5%, respectively (Supplementary Figure S1). Patients with an HJ had considerably longer warm ischemia time (WIT) compared with those who received a DD reconstruction (*p* = 0.001).

**Figure 1:**
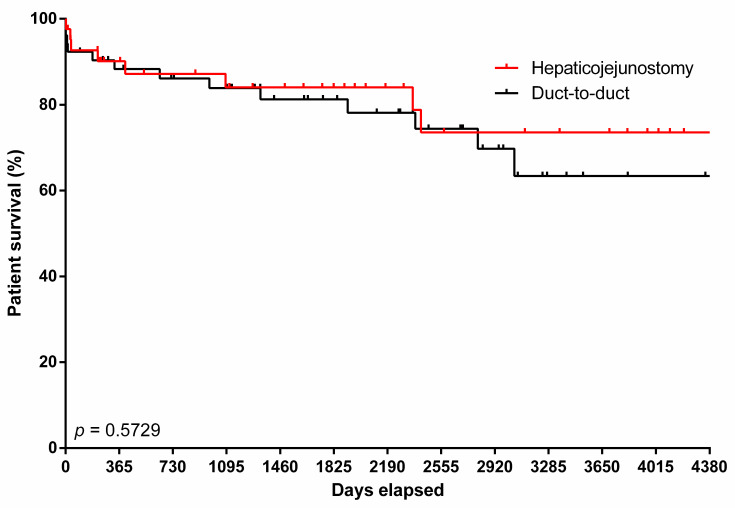
Kaplan–Meier analysis depicting 90-day, 1-year, 3-year, and 5-year survival of patients who underwent liver transplantation for primary sclerosing cholangitis with Roux-en-Y hepaticojejunostomy and duct-to-duct biliary reconstruction.

Patient survival rates in the HJ group at 90 days, 1 year, 3 years, and 5 years were 92.7%, 90.1%, 84.1%, and 84.1%, respectively, compared to 92.3%, 88.3%, 83.9%, and 81.3%, respectively, in the DD group (*p* = 0.573, Figure 1).

### 3.2. Postoperative Complications

Mean postoperative morbidity depicted by the comprehensive complication index, (CCI) as well as the incidence of major postoperative complications, defined as a CCI > 75, showed no difference between the two types of biliary reconstruction (*p* = 0.389 and *p* = 0.412, respectively, Table 2).

Furthermore, there was no difference between the two groups regarding the incidence of bile leakage, episodes of cholangitis, bile duct ischemia, PSC recurrence, or the need for revision surgery because of bile duct complications (*p* = 0.373, *p* = 0.563, *p* = 0.698, *p* = 0.829, and *p* = 0.669, respectively, Table 2). In the DD reconstruction group, five patients received revision surgery because of bile duct-related complications. In four of these patients the complication was anastomotic leakage, while in one patient ischemia of the bile duct was the triggering factor for revision. In two patients, revision of the DD reconstruction was performed, two patients received an HJ, while in the case of one patient a portojejunostomy was carried out. In the HJ group, three patients were surgically revised. Indicating factors were significant stenosis, ischemia of the Roux limb due to an inner hernia, and bile leakage. All patients received a newly performed HJ. However, patients with a DD reconstruction developed anastomotic strictures (30.8% vs. 9.5% in cases of HJ, *p* = 0.012) significantly more often. Notably, the suture technique of DD reconstruction had no impact on the incidence of anastomotic stenosis (33.3% in cases of continuous sutures vs. 16.7% in cases of interrupted sutures, *p* = 0.275, Supplementary Table S6).

### 3.3. Predictors of Mortality

In order to point out independent risk factors of mortality, the Cox proportional hazards model was applied. Variables with a *p* < 0.200 in univariate analysis were included in the Cox regression (Table 3). The MELD score (hazards ratio (HR): 1.098, 95% confidence interval (CI): 1.045–1.155, *p* < 0.001), but not WIT (HR: 1.034, 95% CI: 0.971–1.100, *p* = 0.299), anastomotic strictures (HR: 1.429, 95% CI: 0.431–4.735, *p* = 0.559), or the technique of biliary reconstruction (HR: 1.116, 95% CI: 0.436–2.855, *p* = 0.820), was an independent predictor of mortality.

### 3.4. Risk Factors of Postoperative Complications

In the next phase of our study, we sought to identify risk factors of major postoperative, as well as biliary, complications. Variables with a *p* < 0.200 in univariate analysis were included in multivariate analysis. The MELD score (odds ratio (OR): 1.193, 95% CI: 1.094–1.302, *p* < 0.001) but not the technique of biliary reconstruction (OR: 1.625, 95% CI: 0.390–6.781, *p* = 0.504) or WIT (OR: 0.966, 95% CI: 0.094.1.044, *p* = 0.384) proved to be an independent risk factor of major postoperative complications, defined as a CCI > 75 (Table 4). As for biliary complications, DD reconstruction was identified as an independent predictor of anastomotic strictures (OR: 4.565, 95% CI: 1.265–16.477, *p* = 0.020, Table 5). The MELD score, WIT, or method of biliary reconstruction were not risk factors for bile leakage, cholangitis, PSC-recurrence, bile duct ischemia, or the need for revision surgery because of bile duct complications (*p* = 0.381, *p* = 0.990, *p* = 0.713, *p* = 0.679, and *p* = 0.267, respectively) (Supplementary Tables S1–S5).

## 4. Discussion

Liver transplantation remains the main therapy for PSC, albeit with risks of biliary stenosis and disease recurrence. However, the biliary reconstruction of choice for patients undergoing LT because of PSC is still disputed. Traditionally, HJ reconstruction is preferred over DD reconstruction, based on the hypothesis that the native duct may contribute to the development of anastomotic and non-anastomotic strictures and PSC recurrence.

The recommended type of biliary reconstruction, however, is limited by a paucity of evidence. Existing meta-analyses relied on only 10 studies, all of which were designed retrospectively and were not blinded [9,13].

We present the largest cohort to date of PSC patients undergoing LT in Germany, derived from two high-volume LT centers. Long-term patient survival, as well as general and biliary complications, were analyzed in relation to the type of biliary reconstruction.

The main finding of our study is that a DD reconstruction provides comparable patient survival and postoperative outcomes to the traditional HJ. However, this type of anastomosis is associated with an increased incidence of anastomotic strictures.

There was no difference in 90-day, 1-year, 3-year, and 5-year patient survival between the two groups, which is in line with reports from the US [4], Canada [5], Australia [6], the Netherlands [7], and Iran [8]. Nonetheless, an early publication from the UK, consisting of over 360 patients from seven centers, reported inferior survival in cases of DD reconstruction [3]. However, that study contained patients who were transplanted between 1994 and 2003; since then, general survival after LT increased because of advancements in surgical and organ-preserving techniques, as well as in immunosuppression, making comparison with recent data debatable.

Concerning complications, neither the mean CCI nor the incidence of severe complications, defined as CCI > 75, showed any difference between the two types of biliary reconstruction. The mean CCI in our cohort was 52, which lies slightly above those reported by Schlegel et al. at 9 months postoperative (mean 48.3) [14] or Castanedo et al. (mean 42.4) [15]. It is noteworthy, however, that application of the CCI in the context of LT has been inconsistent across studies [15,16]. A procedure-specific modification of the CCI may therefore be warranted, as has already been demonstrated in pediatric surgery [17,18].

Furthermore, there was no difference between the two groups in our study regarding bile leakage, cholangitis, bile duct ischemia, PSC recurrence, or the need for revision surgery because of bile duct complications. However, patients receiving DD reconstruction exhibited a significantly higher rate of anastomotic strictures compared with those reconstructed via HJ, and DD reconstruction was identified as an independent predictor of anastomotic strictures. This is consistent with data reported by Alwis et al. from Australia [6]. Nonetheless, contradictory data also exist. Sutton et al. found no difference in anastomotic strictures in their cohort. However, in this study, HJ was associated with a higher rate of non-anastomotic strictures as well as a higher incidence of cholangitis [7].

There are two meta-analyses available, which investigated both types of biliary reconstruction in LT for PSC. A meta-analysis of seven studies comparing the two techniques found no significant differences regarding 1-year patient and graft survival, risk of biliary complications, and PSC recurrence [13]. A more recent meta-analysis, containing three additional studies, found no difference between the two types of biliary reconstruction regarding the incidence of strictures, bile leakage, or postoperative mortality rates, while occurrence of cholangitis was higher in cases of HJ [9].

It should be emphasized though, that the higher rate of anastomotic strictures in our study was not followed by inferior survival or a higher incidence of major complications. Furthermore, the type of biliary reconstruction was not an independent predictor of mortality or the incidence of severe postoperative complications. We conclude that the preserved anatomy in cases of DD reconstruction, which facilitates endoscopic interventions like dilatation or stenting, might be the reason for this. These methods have been reported to have an overall success rate of 70–100% in treating such conditions [19].

Overall, PSC recurrence was low in our study (5.3%) and lower than previously reported [10,20]. Neither reconstruction technique, nor MELD score or warm ischemia time were significantly associated with PSC recurrence. One reason for this might be the short follow-up in recently transplanted patients.

Like any other study, our data also had limitations. As a retrospective analysis, it provided less persuasive power than a prospective pragmatic trial. Another factor to consider is the different standards between the two centers represented in our cohort. While the group of patients transplanted at the University Hospital Essen includes LT with both types of biliary reconstruction, all patients transplanted at the University Hospital RWTH Aachen received an HJ, which carries the risk of bias.

Nevertheless, our study possessed several relevant strengths. We report here the largest cohort of PSC patients after LT in Germany from two high-volume centers with specialized LT ICU and high-standard postoperative protocols, which provided an outstanding comparability of all patients.

In conclusion, our data demonstrate equivalent efficacy of DD reconstruction regarding patient survival and postoperative outcomes compared with the traditional HJ. Despite higher rates of anastomotic strictures using this technique, the preservation of an accessible anatomy for endoscopic intervention provides an excellent solution for managing both anastomotic and non-anastomotic strictures, particularly in cases of PSC recurrence. Accordingly, if feasible, a DD anastomosis represents a safe and technically less complex method of biliary reconstruction for PSC patients undergoing LT.

## Figures and Tables

**Table 1:** Demographic, clinical, and donor characteristics of patients who underwent an orthotopic liver transplantation with primary sclerosing cholangitis with biliary reconstruction of either a Roux-en-Y hepaticojejunostomy or a duct-to-duct anastomosis. BMI: body mass index, MELD: model of end-stage liver disease.

	Entire Cohort	Hepaticojejunostomy	Duct-to-Duct Anastomosis	
Characteristic	n = 94	n = 42	n = 52	*p* Value
Age, y, mean (SD)	47.8 (11.9)	49.5 (12.0)	46.4 (11.7)	0.208
Sex, n (%)				
Female	18 (19.0)	10 (23.8)	8 (15.4)	0.302
Male	76 (80.9)	32 (76.2)	44 (84.6)	
MELD score, mean (SD)	14.9 (7.9)	13.5 (7.5)	16.0 (8.1)	0.135
MELD score, n (%)				
<15	54 (57.4)	26 (61.9)	28 (53.8)	
15–20	22 (23.4)	10 (23.8)	12 (23.1)	
21–25	9 (9.6)	3 (7.1)	6 (11.5)	
26–30	2 (2.1)	0 (0.0)	2 (3.8)	
>30	7 (7.4)	3 (7.1)	4 (7.7)	
BMI, kg/m^2^, mean (SD)	24.2 (4.2)	24.1 (4.1)	24.3 (4.2)	0.657
BMI, n (%)				
<18.5	3 (3.2)	1 (2.4)	2 (3.8)	
18.5–24.9	59 (62.8)	27 (64.3)	32 (61.5)	
25–29.9	25 (26.6)	11 (26.2)	14 (26.9)	
30–34.9	6 (6.4)	3 (7.1)	3 (5.8)	
≥35	1 (1.1)	0 (0.0)	1 (1.9)	
Transplant center, n (%)				
Aachen	21 (22.3)	21 (50.0)	0 (0.0)	<0.001
Essen	73 (77.7)	21 (50.0)	52 (100.0)	
Donor Age, y, mean (SD)	52.6 (17.6)	51.2 (16.9)	53.7 (18.1)	0.500
Donor Sex, n (%)				
Female	36 (38.3)	18 (42.9)	18 (34.6)	0.414
Male	58 (61.7)	24 (57.1)	34 (65.4)	
Donor BMI, kg/m^2^, mean (SD)	27.2 (5.5)	27.5 (6.3)	26.9 (4.9)	0.676
Warm ischemia time, min (SD)	34 (7)	37 (7)	32 (7)	0.001
Cold ischemia time, h (SD)	7.7 (1.8)	7.8 (2.0)	7.6 (1.6)	0.483

**Table 2:** General postoperative and biliary complications after orthotopic liver transplantation with primary sclerosing cholangitis with biliary reconstruction of either a Roux-en-Y hepaticojejunostomy or a duct-to-duct anastomosis. CCI: comprehensive complication index, PSC: primary sclerosing cholangitis.

	Entire Cohort	Hepaticojejunostomy	Duct-to-Duct Anastomosis	
Characteristic	n = 94	n = 42	n = 52	*p* Value
CCI, mean (SD)	52 (34)	48 (34)	54 (34)	0.389
CCI > 75, n (%)	24 (25.5)	9 (22.4)	15 (28.8)	0.412
Anastomotic stricture, n (%)	20 (21.3)	4 (9.5)	16 (30.8)	0.012
Bile leakage, n (%)	7 (7.4)	2 (4.8)	5 (9.6)	0.373
Episode of cholangitis, n (%)	6 (6.4)	2 (4.8)	4 (7.7)	0.563
Biliary duct ischemia, n (%)	3 (3.2)	1 (2.4)	2 (3.8)	0.688
PSC recurrence, n (%)	5 (5.3)	2 (4.8)	3 (5.8)	0.829
Revision surgery because of bile duct complication, n (%)	8 (8.5)	3 (7.1)	5 (9.6)	0.669

**Table 3:** Cox proportional hazards model for patient survival. MELD: model of end-stage liver disease.

Variable	HR (95% CI)	*p* Value
Biliary Reconstruction Technique	1.116 (0.436–2.855)	0.820
Anastomotic Stricture	1.429 (0.431–4.735)	0.559
MELD Score	1.099 (1.045–1.155)	<0.001
Warm Ischemia Time	1.034 (0.971–1.100)	0.299

**Table 4:** Logistic regression for major postoperative complications, defined as a CCI > 75. CCI: comprehensive complication index, MELD: model of end-stage liver disease.

Variable	OR (95% CI)	*p* Value
MELD Score	1.193 (1.094–1.302)	<0.001
Biliary Reconstruction Technique	1.627 (0.390–6.781)	0.504
Warm Ischemia Time	0.966 (0.094–1.044)	0.384

**Table 5:** Logistic regression for anastomotic stricture. MELD: model of end-stage liver disease.

Variable	OR (95% CI)	*p* Value
MELD Score	0.932 (0.857–1.014)	0.101
Biliary Reconstruction Technique	4.565 (1.265–16.477)	0.020
Warm Ischemia Time	0.984 (0.920–1.054)	0.650

## Data Availability

Datasets analyzed during the current study are not publicly available due to patient privacy limitations, but are available from the corresponding author on reasonable request.

## Supplementary Materials

The following supporting information can be downloaded at: https://www.mdpi.com/article/10.3390/jcm14238518/s1, Supplementary Figure S1: Overall Survival of the whole cohort of patients who underwent liver transplantation for primary sclerosing cholangitis. Supplementary Table S1. Logistic regression for bile leakage. MELD: model of end-stage liver disease. Supplementary Table S2. Logistic regression for cholangitis MELD: model of end-stage liver disease. Supplementary Table S3. Logistic regression for PSC recurrence. MELD: model of end-stage liver disease, PSC: primary sclerosing cholangitis. Supplementary Table S4. Logistic regression for bile duct ischemia. MELD: model of end-stage liver disease. Supplementary Table S5. Logistic regression for revision surgery because of bile duct complication. MELD: model of end-stage liver disease. Table S6. Biliary complications after orthotopic liver transplantation for primary sclerosing cholangitis with a duct-to-duct biliary reconstruction with either continuous or interrupted sutures. Seven patients were excluded due to lack of data regarding the type of suture of the duct-to-duct anastomosis. PSC: primary sclerosing cholangitis.

## Abbreviations

The following abbreviations are used in this manuscript:

CCI	Comprehensive Complication Index
CI	Confidence Interval
DD	Duct-to-Duct Reconstruction
ET	Eurotransplant
HJ	Roux-en-Y Hepaticojejunostomy
HR	Hazards Ratio
LT	Liver Transplantation
MELD	Model of End-Stage Liver Disease
OR	Odds Ratio
PSC	Primary Sclerosing Cholangitis
WIT	Warm Ischemia Time
