# Supplementary material for: Biliary Reconstruction in Liver Transplantation with Primary Sclerosing Cholangitis: Roux-en-Y Hepaticojejunostomy or Duct-to-Duct Anastomosis?
Source: J Clin Med. 2025 Dec 1;14(23):8518. doi: 10.3390/jcm14238518 (PMC12693685; doi:10.3390/jcm14238518)
Supplement: Supplementary file 1 [file jcm-14-08518-s001.zip › Supplementary Tables.pdf]

Variable	OR (95% CI)	*p* value
MELD Score	0.993 (0.898-1.098)	0.398
Biliary Reconstruction Technic	2.258 (0.366-13.943)	0.381
Warm Ischemia Time	0.993 (0.898-1.098)	0.890

Supplementary Table S1. Logistic regression for bile leakage. MELD: model of end-stage liver disease.

Variable	OR (95% CI)	*p* value
MELD Score	1.006 (0.911-1.112)	0.904
Biliary Reconstruction Technic	0.987 (0.147-6.625)	0.990
Warm Ischemia Time	0.925 (0.836-1.023)	0.127

Supplementary Table S2. Logistic regression for cholangitis MELD: model of end-stage liver disease.

Variable	OR (95% CI)	*p* value
MELD Score	0.856 (0.681-1.077)	0.186
Biliary Reconstruction Technic	1.465 (0.191-11.235)	0.713
Warm Ischemia Time	0.991 (0.880-1.117)	0.886

Supplementary Table S3. Logistic regression for PSC recurrence. MELD: model of end-stage liver disease, PSC: primary sclerosing cholangitis.

Variable	OR (95% CI)	*p* value
MELD Score	1.019 (0.890-1.168)	0.782
Biliary Reconstruction Technic	1.725 (0.130-22.859)	0.679
Warm Ischemia Time	1.023 (0.880-1.188)	0.770

Supplementary Table S4. Logistic regression for bile duct ischemia. MELD: model of end-stage liver disease.

Variable	OR (95% CI)	*p* value
MELD Score	0.991 (0.892-1.101)	0.865
Biliary Reconstruction Technic	2.808 (0.454-17.373)	0.267
Warm Ischemia Time	1.051 (0.946-1.167)	0.354

Supplementary Table S5. Logistic regression for revision surgery because of bile duct complication. MELD: model of end-stage liver disease.

Characteristic	Entire Duct-to-Duct Anastomosis cohort<br>n = 45	Continuous sutures<br>n = 33	Interrupted sutures<br>n = 12	*p* value
Anastomotic stricture, n (%)	13 (28.9)	11 (33.3)	2 (16.7)	0.275
Bile leakage, n (%)	5 (11.1)	5 (15.1)	0 (0.0)	0.153
Episode of cholangitis, n (%)	4 (8.9)	3 (9.1)	1 (8.3)	0.937
Biliary duct ischemia, n (%)	2 (4.4)	1 (3.0)	1 (8.3)	0.445
PSC recurrence, n (%)	5 (11.1)	4 (12.1)	1 (8.3)	0.721
Revision surgery because of bile duct complication, n (%)	1 (2.2)	1 (3.0)	0 (0.0)	0.542

Supplementary Table S6. Biliary complications after orthotopic liver transplantation for primary sclerosing cholangitis with a duct-to-duct biliary reconstruction with either continuous or interrupted sutures. Seven patients were excluded due to lack of data regarding the type of suture of the duct-to-duct anastomosis. PSC: primary sclerosing cholangitis.

Supplementary Figure Legend:

Supplementary Figure S1: Overall Survival of the whole cohort of patients who underwent liver transplantation for primary sclerosing cholangitis.
