# Supplementary figures and images for: Biliary Reconstruction in Liver Transplantation with Primary Sclerosing Cholangitis: Roux-en-Y Hepaticojejunostomy or Duct-to-Duct Anastomosis?
Source: J Clin Med. 2025 Dec 1;14(23):8518. doi: 10.3390/jcm14238518 (PMC12693685; doi:10.3390/jcm14238518)

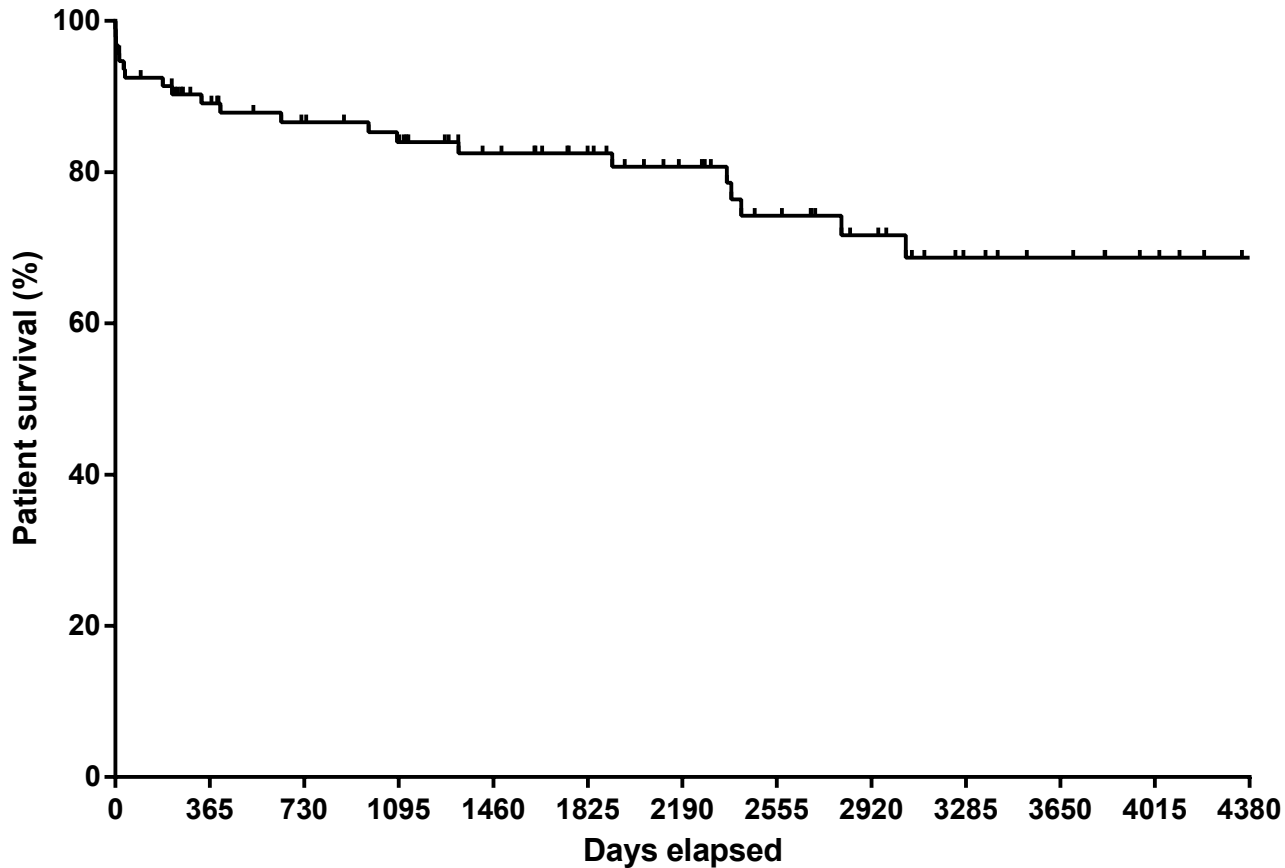

Supplement: Supplementary file 1 [file jcm-14-08518-s001.zip › Supplementary Figure S1.pdf]
